# Bilateral Congenital Hypoplasia of the Extensor Tendons of the Hands

**DOI:** 10.7759/cureus.79698

**Published:** 2025-02-26

**Authors:** Dalton Beeson, Benjamin D Brooks, Thomas Bigham

**Affiliations:** 1 Department of Primary Care Medicine, Rocky Vista University College of Osteopathic Medicine, Ivins, USA; 2 Department of Biomedical Sciences, Rocky Vista University College of Osteopathic Medicine, Ivins, USA

**Keywords:** congenital hypoplasia, extensor tendons, hand function, rare, tendon transfer

## Abstract

Congenital hypoplasia or aplasia of the extensor tendons of the hands is an extremely rare condition characterized by an inability to extend the fingers due to underdeveloped or absent extensor tendons. This case study presents a 26-year-old female born with the inability to extend digits 2-5 bilaterally, an anomaly that has significantly impacted her hand function throughout her life. Despite multiple medical evaluations, tendon transfer surgery, and physical and occupational therapy, the patient has experienced mixed outcomes, with marginal improvement in some areas and persistent functional limitations in others. The rarity of this condition poses significant challenges in diagnosis and management, and treatment options must be highly individualized. This case highlights the complexities of managing congenital tendon anomalies and reinforces the need for more research to improve treatment outcomes.

## Introduction

Congenital hypoplasia or aplasia of the extensor tendons of the hands is a rare musculoskeletal anomaly characterized by the inability to extend the fingers. This condition arises from the underdevelopment or absence of extensor tendons, leading to significant impairment in hand function and fine motor tasks. While more commonly affecting the thumb, isolated cases involving the inability to extend digits 2-5, known as congenital hypoplasia/aplasia of the extensor tendons, are exceedingly rare, with only a few documented cases worldwide [1,2]. The extensor digitorum, the primary muscle responsible for extending digits 2-5 at the metacarpophalangeal (MCP), proximal interphalangeal (PIP), and distal interphalangeal (DIP) joints, plays a crucial role in hand function. Deficiencies in this muscle or its tendons can result in substantial functional limitations [3,4]. The exact cause of this condition remains unclear.

Given the rarity of this condition, the literature is primarily limited to isolated case reports, often describing varying treatment courses and outcomes. Management strategies are often trial-and-error, as there is no established treatment protocol for this condition. This case study discusses a 26-year-old female with a congenital bilateral inability to extend digits 2-5, highlighting the challenges in diagnosis, management, and treatment options for this rare condition.

## Case presentation

A 26-year-old female presents with the inability to fully extend most of her fingers since birth. Past medical history includes depression and anxiety. Family history is significant for rheumatoid arthritis in her mother. The thumbs of both hands are unaffected. Findings are worse on the left hand. Associated symptoms include bilateral mild weakness and decreased grip strength. The patient denies pain, numbness, or tingling. The patient is right-hand dominant. The patient’s condition affects her everyday life, but mostly in activities requiring repetitive extension of the fingers in fine motor movements, such as typing, using scissors, writing, and playing musical instruments. 

**Figure 1:**
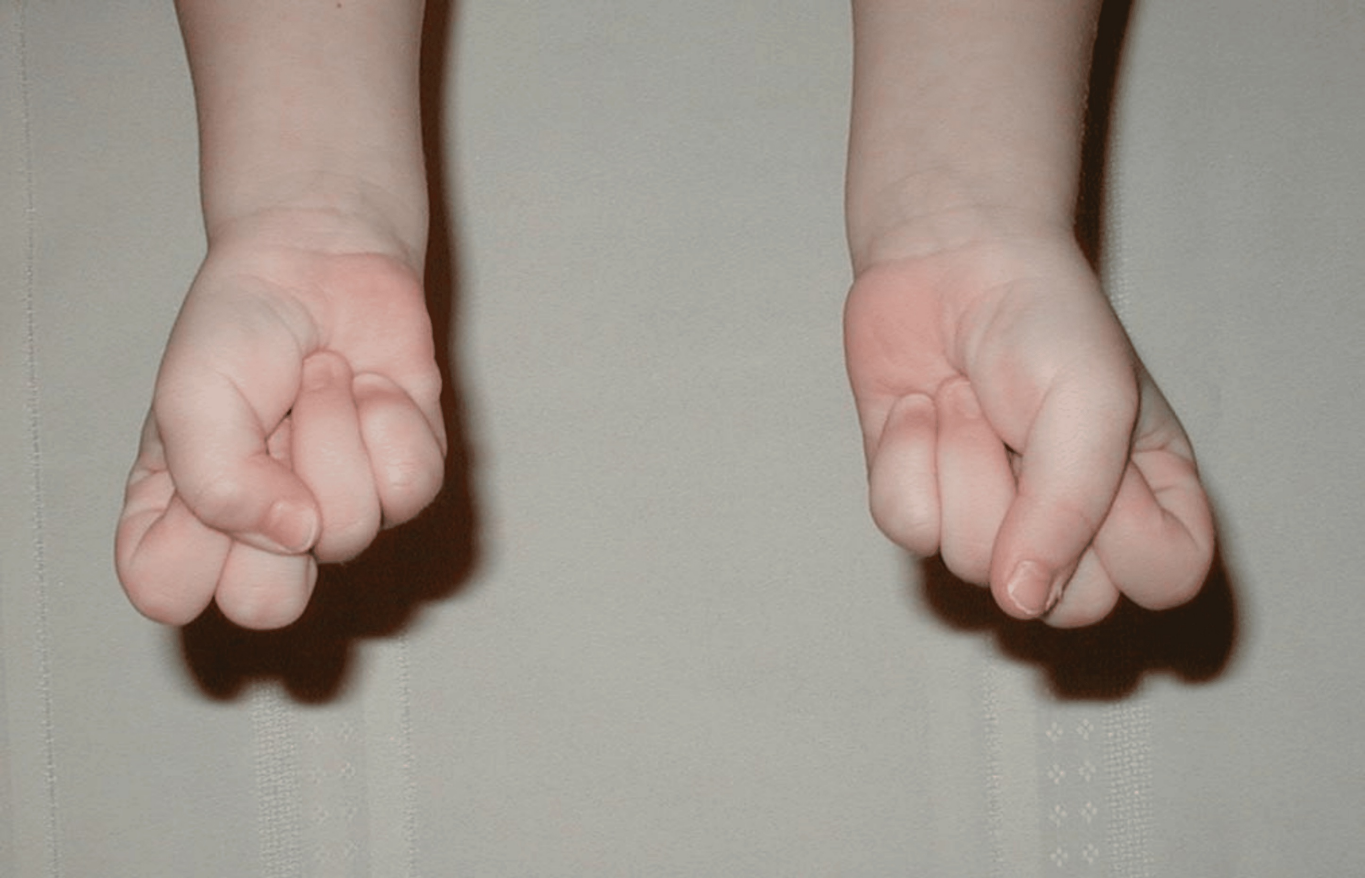
Patient's hands at age 3 (view 1).

**Figure 2:**
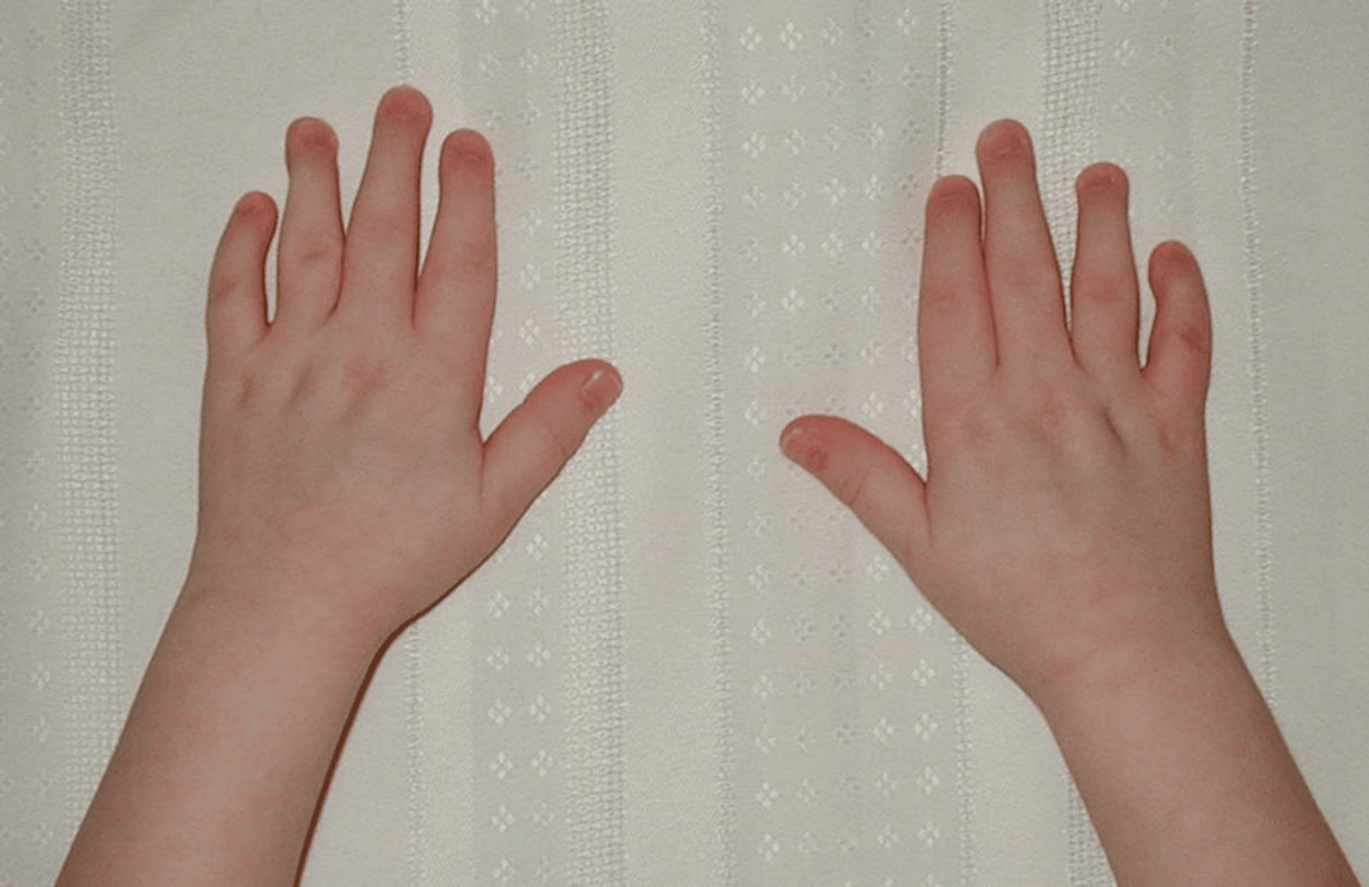
Patient's hands at age 3 (view 2).

**Figure 3:**
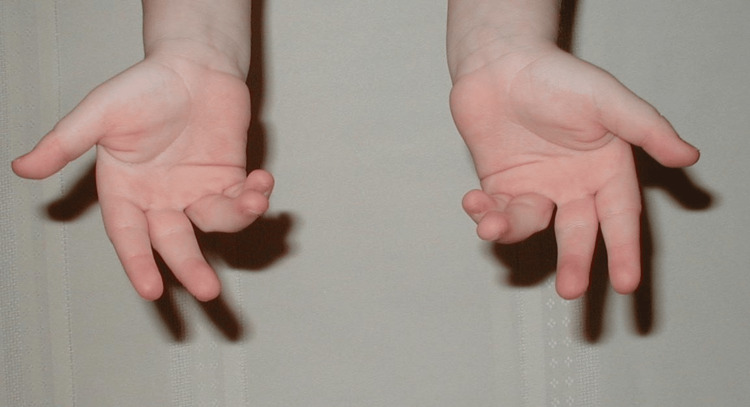
Patient's hands at age 3 (view 3).

At birth, the patient presented with flexed fingers 2-5, extended thumbs, and an inability to open-fisted hands. No other abnormalities were noted. The patient was born full-term via spontaneous vaginal delivery to healthy parents, and newborn screening was unremarkable. The patient did not crawl and started walking at eight months. All other developmental milestones were met without deficiency. Throughout childhood, this patient was evaluated by many medical professionals, including pediatricians and hand specialists, who recommended regular monitoring but no specific treatment due to the patient’s age, lack of other concerning findings, and unknown diagnosis and management of this condition. Figures 1-3 show photos of the patient's hands at age three, which were provided by the patient.

At age 12, the patient was referred to a pediatric hand surgeon to discuss treatment options for her persistent inability to extend digits 2-5 bilaterally. On exam, the patient was able to maintain full extension of the digits for a few seconds after passive extension but was unable to perform active extension. Findings were worse on digits 4 and 5 of the right hand. Associated symptoms included a soft tissue contracture of the palmar surface of the right fourth finger and mild hand weakness. X-rays of both hands showed no significant abnormalities. MRI of the right hand showed the absence of the extensor digiti minimi, extensor digitorum, and extensor indices muscles and tendons; anomalous extensor muscles arising from the level of metacarpals at the second and third digits; and normal extensor carpi ulnaris, extensor hallucis longus, extensor carpi radialis longus and brevis, abductor hallucis longus, and extensor hallucis brevis muscles and tendons. 

Based on the MRI findings, a tendon transfer surgery was performed; the right flexor digitorum superficialis tendon of the third digit was transferred to the extensor surfaces of the fourth and fifth digits on the right hand. This was followed by a corrective surgery to readjust the tightness of the tendon. Outpatient physical therapy was performed for six weeks post-surgery. Six weeks post-surgery, the patient reported improved extension and strength in all joints of the right fourth and fifth digits but decreased flexion at the right fourth and fifth metacarpophalangeal (MCP) joints due to increased tendon tightness and a buildup of scar tissue. A swan neck deformity of the right third digit was observed due to the removal of the FDS. Slight extension deficits in digits 2 and 3 on the right were unchanged. Right-hand grip strength was decreased due to the reduced flexion and buildup of scar tissue. Due to these mixed results, the patient did not opt for tendon transfer of the left hand. 

At age 16, the patient participated in occupational therapy, including serial casting for six weeks, to address the inability to fully flex the fourth and fifth digits on her right hand. The casting did not result in significant improvement. 

**Figure 4:**
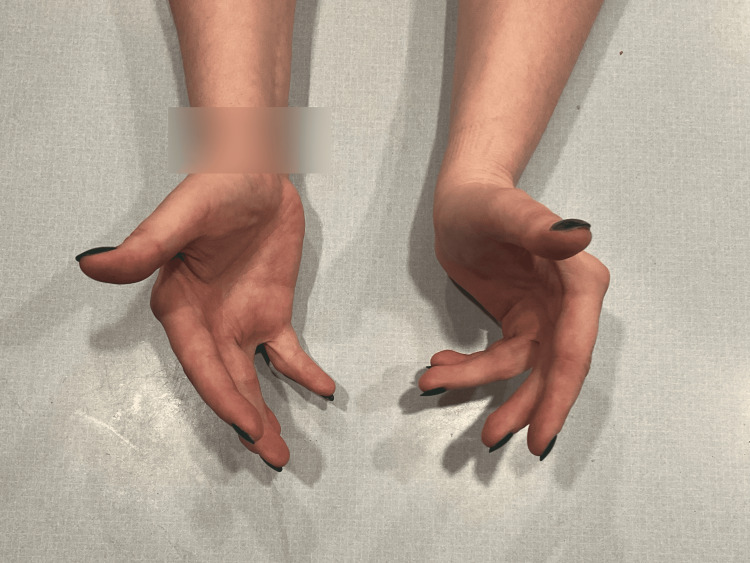
Patient's hands-on presentation (age 26) (view 1).

**Figure 5:**
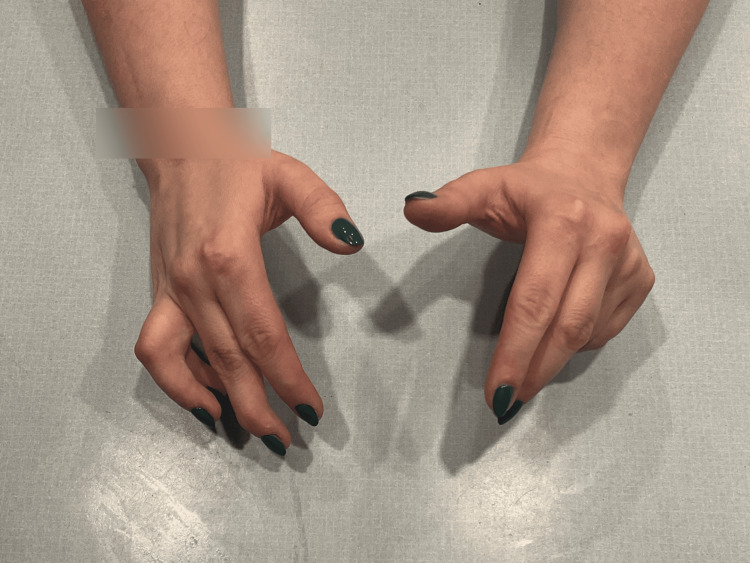
Patient's hands-on presentation (age 26) (view 2).

**Figure 6:**
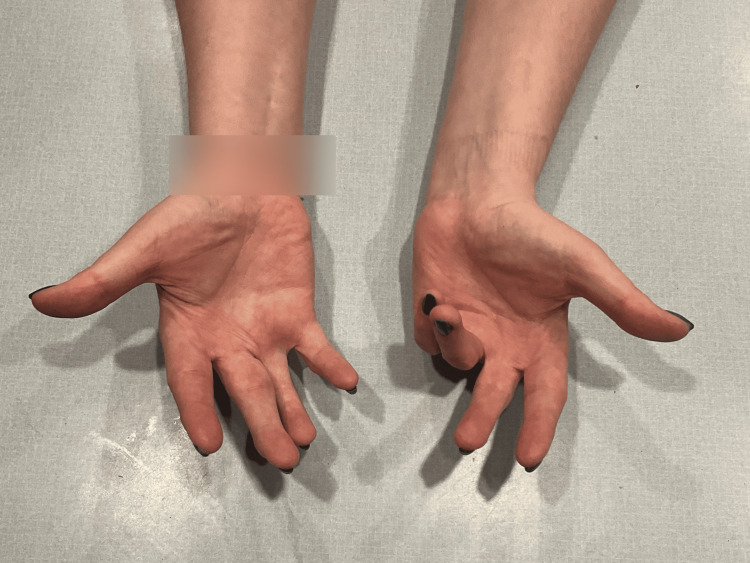
Patient's hands-on presentation (age 26) (view 3).

**Figure 7:**
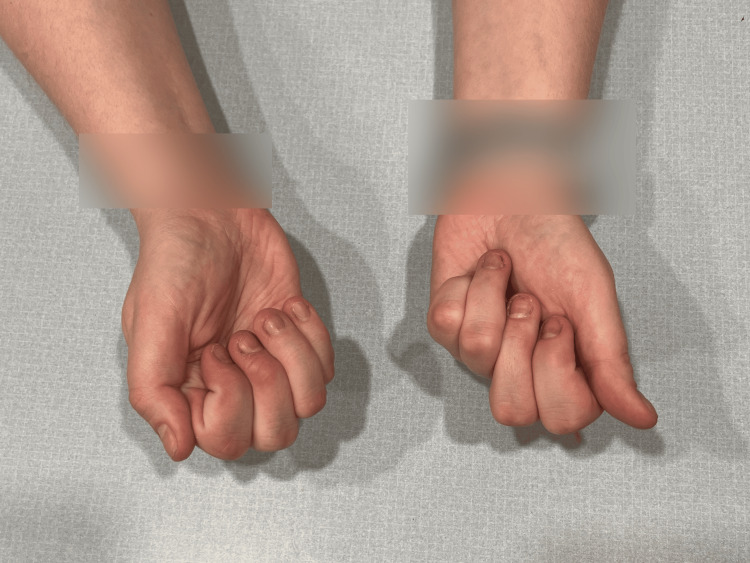
Patient's hands-on presentation (age 26) (view 4).

On the current physical exam, the patient has a varying ability to extend each finger. On the left hand, digits 2 and 3 are unable to extend at the MCP joint fully but have full extension at the proximal interphalangeal (PIP) and distal interphalangeal (DIP) joints. Left-hand digits 4 and 5 are unable to fully extend at the MCP, DIP, and PIP joints, presenting similar to an ulnar claw deformity. On the right hand, digits 2 and 3 are unable to fully extend at the MCP joint but have full extension at the PIP and DIP joints. Right-hand digits 4 and 5 have a full extension at the MCP, PIP, and DIP joints but are unable to fully flex at the MCP joints. A swan neck deformity is present on digit 3 on the right hand. Grip strength is 5/5 bilaterally. No sensory or neurological deficits are noted. Other upper extremity strength and motion testing are unremarkable. Figures 4-7 show photos of the patient's hands-on presentation (age 26). 

While no definitive diagnosis was ever presented to the patient by physicians, likely due to the rarity of the condition, through her own investigation, she inferred her condition to be bilateral congenital hypoplasia of the extensor tendons of the hands. This inference was based on the MRI results and the patient’s research of the few similar cases available in the literature. 

## Discussion

This case study discusses a 26-year-old female with a congenital bilateral inability to extend digits 2-5, highlighting the challenges in diagnosis, management, and treatment options for this rare condition. Congenital hypoplasia of the extensor tendons is a rare and complex condition, with fewer than 20 documented cases affecting multiple digits [1,4]. Most reported cases focus on the thumb, with very few involving digits 2-5 [2]. The rarity of this condition makes diagnosis and treatment particularly challenging, as the literature is primarily limited to isolated case reports with limited data available to guide workup and management.

The exact etiology of congenital hypoplasia of the extensor tendons remains unknown. Various hypotheses have been proposed, including developmental failure during the myoblastic stage or lack of radial nerve innervation during fetal development [2,5]. Some cases involve a complete absence of both flexor and extensor tendons in one digit, further illustrating the variability of this condition’s presentation [6]. Additionally, some cases of congenital extensor tendon hypoplasia have been genetically linked, with both autosomal dominant and autosomal recessive inheritance patterns being reported [3,4]. While the potential for genetic inheritance exists, it is uncertain whether this patient could pass this condition to her offspring, as she has no family history of this condition. Genetic counseling could be considered for future family planning [6]. 

Due to the rarity of congenital hypoplasia, surgical management is typically specific to each case, with tendon transfers being one of the primary options. In this patient’s case, a flexor digitorum superficialis tendon transfer was performed. Several other tendons have been historically used in surgical repairs with varying success [2]. The extensor carpi radialis longus is frequently utilized, particularly for restoring function in multiple digits [3,4]. Other tendons, such as the flexor carpi radialis and palmaris longus, have also been reported in the literature, with outcomes depending on the specific tendons transferred and the patient’s overall anatomy [1,4,7]. While these surgeries can improve functionality, the outcomes often yield mixed results, as in this case. Although the patient reported improved extension and strength in all joints of the right fourth and fifth digits after the right FDS tendon transfer, she did not opt for a tendon transfer of the left hand due to the decreased grip strength, decreased flexion at the right fourth and fifth MCP joints, and swan neck deformity as a result of the surgery. 

Physical therapy is a key component of post-surgical rehabilitation and conservative management of congenital extensor tendon hypoplasia. In many cases, intensive therapy following surgery has been shown to enhance the recovery of function. For example, in one case, a 12-year-old boy regained a significant range of motion in the middle and ring fingers after six months of post-operative physical therapy following tendon transfer surgery [1]. Similarly, prolonged and targeted postsurgical rehabilitation has been deemed to be crucial for optimal results in such rare conditions [8]. By contrast, in this patient, six weeks of post-operative physical therapy led to mixed results, suggesting that a longer or more structured rehabilitation period may be necessary [1]. 

In addition, occupational therapy, including splinting or serial casting, could be pursued as conservative treatment or as an adjunct to post-surgical care. However, the efficacy of this intervention has not been reported in the literature in individuals with this rare diagnosis. In this case, the patient underwent serial casting at age 16 to address the inability to flex the fourth and fifth digits of her right hand, but the intervention did not result in significant improvement. This outcome highlights the variability in the efficacy of conservative treatments and underscores the need for additional research to be completed in regard to occupational therapy and congenital tendon hypoplasia. 

## Conclusions

Bilateral congenital hypoplasia of the extensor tendons of the hands is an exceedingly rare condition that presents numerous challenges in both diagnosis and management. The lack of a standardized treatment protocol combined with the rarity of the condition means that treatment must be highly individualized, considering patient anatomy and functional goals. While tendon transfers remain the mainstay of surgical management, outcomes may vary, and additional therapies, such as physical and occupational therapy, play a crucial role in enhancing functionality. Further research and case studies such as this are needed to explore the efficacy of both conservative and surgical therapy for the treatment of this condition and ultimately improve functional outcomes for future patients.

## References

[1] Tungshusakul S, Leechavengvongs S, Uerpairojkit C (2011). Bilateral congenital hypoplasia of the extensor tendons of the hand: a case report. Hand Surg.

[2] Vartanian ED, Cohen MJ, Kulber DA (2020). Congenital hypoplasia of the extensor tendons of the fingers: a case report and review of the literature. J Hand Surg Am.

[3] Vartany A, Majumdar S, Diao E (1996). Congenital hypoplasia of the extensor tendons of the hand. J Hand Surg Am.

[4] Wajid MA, Rangan A (2001). Congenital aplasia or hypoplasia of extensor tendons of the hand--a case report and review of the literature. J R Coll Surg Edinb.

[5] Zadek I (1934). Congenital absence of the extensor pollicis longus of both thumbs. Operation and cure. J Bone Joint Surg Am.

[6] Jerome JTJ (2024). Congenital absence of flexor and extensor tendons in the middle finger: a rare disorder. J Musculoskelet Surg Res.

[7] Aivaz M, Mantilla-Rivas E, Brennan A (2023). Bilateral digital extensor hypoplasia correction: a case report and systematic review. Arch Plast Surg.

[8] Yammine K (2015). The prevalence of the extensor indicis tendon and its variants: a systematic review and meta-analysis. Surg Radiol Anat.

